# Probing orthobunyavirus reassortment using Bunyamwera and Batai viruses as models

**DOI:** 10.1371/journal.pntd.0013120

**Published:** 2025-05-30

**Authors:** James M. Bowen, Krista Gunter, Andrew M. Lunel, Dorcus C. A. Omoga, Jennifer E. Jones, Henry Giesel, W. Paul Duprex, Natasha L. Tilston

**Affiliations:** 1 Department of Microbiology and Immunology, Indiana University School of Medicine, Indianapolis, Indiana, United States of America; 2 Department of Pediatrics, Herman B Wells Center for Pediatric Research, Indiana University School of Medicine, Indianapolis, Indiana, United States of America; 3 Department of Pediatrics, University of Pittsburgh School of Medicine, Pittsburgh, Pennsylvania, United States of America; 4 Center for Vaccine Research, School of Medicine, University of Pittsburgh, Pittsburgh, Pennsylvania, United States of America; 5 Department of Microbiology and Molecular Genetics, School of Medicine, University of Pittsburgh, Pittsburgh, Pennsylvania, United States of America; Texas A&M University, UNITED STATES OF AMERICA

## Abstract

Reassortment is a critical evolutionary mechanism for segmented viruses, enabling the exchange of intact genome segments during co-infection and driving orthobunyavirus evolution; however, the molecular mechanisms underpinning this process remain unclear. With over 100 orthobunyavirus species, many of which are significant human and veterinary pathogens, understanding how reassortment influences transmissibility and virulence is essential for preempting the emergence of novel pathogens. Here, we use Bunyamwera virus (BUNV) and Batai virus (BATV) as models to explore orthobunyavirus reassortment through reverse genetics. We established the first reverse genetics system for BATV, generated reassortants, and employed minigenome assays to assess replication machinery compatibility. Additionally, we developed a novel hybridization chain reaction assay for high-resolution visualization of viral RNA segments. Our findings revealed that all six reassortants between BUNV and BATV are viable, exhibiting notable phenotypic differences in interferon-deficient (IFNAR^-/-^) mice. This work introduces essential tools and new insights into orthobunyavirus reassortment and pathogenesis, laying the groundwork for understanding this critical evolutionary process.

## Author summary

Orthobunyaviruses are a diverse group of segmented RNA viruses responsible for severe diseases in humans and animals. Despite their potential to cause significant public health and veterinary outcomes, the molecular mechanisms driving their evolution through reassortment remain largely unexplored. This study leverages Bunyamwera virus (BUNV) and Batai virus (BATV) as models to investigate reassortment dynamics. Our comprehensive approach, combining reverse genetics, pathogenesis, and a novel RNA-FISH assay, demonstrated the viability of all six possible BUNV-BATV reassortants and revealed distinct pathogenic profiles in interferon-deficient mice. These findings are critical for understanding how reassortment can alter viral transmissibility and virulence, offering valuable tools and insights for future studies. Understanding the reassortment process is essential for anticipating and mitigating the risk posed by novel reassortant viruses.

## Introduction

RNA viruses exhibit remarkable diversity due to their inherently unstable genomes. The lack of a proofreading polymerase and rapid replication cycles, coupled with their ability to undergo recombination and reassortment, further amplify this diversity [1]. Recombination merges distinct genome sections to form chimeric genomes from co-infecting viruses, while reassortment, unique to segmented viruses, allows the exchange of entire genome segments [2]. This process preserves intact genetic information and can drive substantial evolutionary shifts, potentially altering the pathogenicity and transmissibility of the resulting progeny (reassortants) [3]. While reassortment is well-documented in influenza viruses, where it contributes to the emergence of pandemic strains [3,4], its role in bunyaviruses remains understudied. This gap is particularly concerning as bunyaviruses are associated with severe human and veterinary diseases, including hemorrhagic fever, neurological conditions, and various teratogenic outcomes [5,6]. Notably, Rift Valley fever virus and Crimean-Congo hemorrhagic fever virus are classified as priority pathogens by the World Health Organization [7], while the National Institutes of Health (NIH) has included several bunyaviruses in its pandemic preparedness network, highlighting the urgent need for targeted research [8].

Amongst bunyaviruses, the genus *Orthobunyavirus* within the *Peribunyaviridae* family represents the largest group of arthropod-transmitted pathogens [5,9]. These viruses possess a single-stranded, tripartite, negative-sense RNA genome consisting of a Small (S), Medium (M), and Large (L) segment. The S segment encodes the nucleoprotein (N), with some viruses also producing a non-structural protein (NSs) via an overlapping open reading frame. The M segment encodes a polyprotein that is co-translationally cleaved into glycoproteins (Gn and Gc) and a non-structural protein (NSm), and the L segment encodes the RNA-dependent RNA polymerase (RdRp). The untranslated regions (UTRs) flanking these coding sequences contain essential transcription, replication, and packaging signals. The N protein encapsidates the viral (v) RNA and, along with the RdRp, forms the ribonucleoprotein complex (RNP). These RNPs are trafficked to the Golgi apparatus, where they are packaged with glycoproteins for virus maturation and release. This packaging process is believed to be loosely regulated, which may increase the likelihood of segment mispackaging and promote reassortment [5,9,10].

Over the years, numerous orthobunyavirus reassortants have been identified [2], with Ngari virus (NRIV) serving as a compelling example of reassortment’s impact on human and veterinary health [11–15]. NRIV emerged through the reassortment of Bunyamwera virus (BUNV) S and L segments and Batai virus (BATV) M segment. BUNV and BATV share a relatively close genetic relationship and a geographic overlap in Africa. While BUNV and BATV typically cause mild, self-limiting illnesses in humans and primarily affect ruminants, NRIV is associated with human hemorrhagic fever. The mechanisms driving this drastic phenotypic shift remain unclear. First detected in the 1970s, NRIV has since spread across sub-Saharan Africa, presenting a significant threat to both public and veterinary health [15]. This emergence emphasizes the critical need to understand the molecular and cellular mechanisms driving orthobunyavirus reassortment to preempt the rise of such novel and highly pathogenic viruses.

To address this, we have developed model systems using BUNV and BATV. Here, we present the development of (a) minigenome assays to evaluate the compatibility of BUNV and BATV transcription/replication machinery, demonstrating the utility of these assays in reassortment studies; (b) the first rescue system for BATV, enabling the generation of recombinant (r) BUNV and BATV reassortants; and (c) a novel multiplex single-molecule hybridization chain reaction (HCR) assay and custom-built image analysis pipeline, allowing precise, high-resolution visualization and quantification of individual vRNA segments. Using these systems, we demonstrate the flexible nature of BUNV and BATV reassortment, showing that all six reassortment combinations are viable despite only one reassortant being observed in nature (i.e., NRIV). Furthermore, we show that BUNV and BATV induce distinct disease phenotypes in interferon (IFN) α/β receptor knock-out (IFNAR^-/-^) mice, with the M segment reassortants (including NRIV-like virus) producing intermediate phenotypes. The tools and insights presented here establish a robust framework for characterizing BUNV, BATV, and their reassortants while providing a critical foundation to study the genomic drivers of segment exchange, viral fitness, and virulence. With the rising ecological changes heightening the risk of arbovirus transmission [16], this study provides crucial tools to evaluate the threats posed by orthobunyavirus reassortment.

## Results

### The N and RdRp of rBUNV and rBATV are compatible with each other’s minigenomes

To assess the reassortment potential between BUNV and BATV, we utilized a minigenome assay, a proven method for evaluating transcription and replication compatibility among orthobunyaviruses [17]. To do this, we constructed minigenome plasmids encoding fluorescent reporters dTomato, TagBFP, and Venus flanked by the full-length UTRs of the S, M, and L genome segments, respectively, of either BUNV, previously described [18], or BATV (strain MM2222). These minigenome plasmids enabled us to evaluate whether the N and RdRp proteins from rBUNV and rBATV could recognize and initiate transcription and replication of each other’s (heterologous) minigenomes.

**Fig 1:**
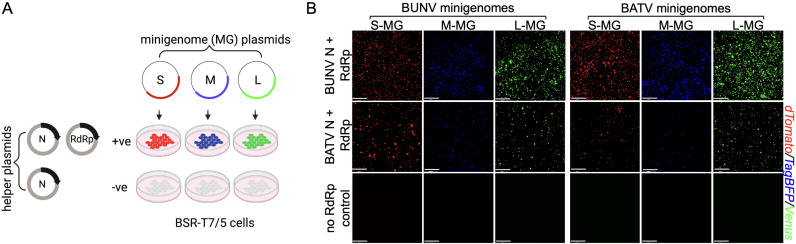
BUNV and BATV minigenome assay. (A) Schematic of a minigenome assay (created in BioRender. Tilston, N. (2025) https://BioRender.com/9ry0hhq). BSR-T7/5 cells transfected with plasmids expressing minigenomes along with N and RdRp expression plasmids. Minigenome transcripts are expressed in the negative sense and require both N and RdRp for transcription and replication. (B) BUNV and BATV minigenomes. BSR-T7/5 transfected with S, M, or L minigenome plasmids along with either BUNV or BATV N and RdRp expression plasmids. Controls contained minigenome and N expression plasmids only. 2 days post-transfection, cells were visualized using a fluorescence microscope. Representative images are shown out of an n = 3. Scale bar 534.9 μm.

Following transfection of BSR-T7/5 cells with the minigenome plasmids alongside homologous or heterologous N and RdRp helper plasmids (Fig 1A), we observed efficient transcription and replication of minigenomes from both rBUNV and rBATV. Robust fluorescent signals were detected for all three minigenomes (S, M, and L segments) when co-transfected with either homologous or heterologous N and RdRp expression plasmids, confirming functional compatibility between the N and RdRp complexes of rBUNV and rBATV (Fig 1B). Notably, a lower proportion of fluorescent cells was observed in assays utilizing BATV N + RdRp compared to BUNV N + RdRp. This difference may stem from the use of different expression vectors, pTM1 (T7-driven) for BUNV versus pCG (CMV-driven; human cytomegalovirus immediate-early promoter) for BATV. Since the purpose of the assay was to qualitatively assess reporter activity rather than provide quantitative measurements, this discrepancy was deemed acceptable for the scope of this study. Overall, these results reveal that the N and RdRp complexes of rBUNV and rBATV are functionally interchangeable. This suggests that reassortment between BUNV and BATV may occur across a broader range of segment combinations than previously observed, extending beyond the naturally occurring NRIV.

### All six reassortant combinations between rBUNV and rBATV are viable in vitro

To validate the reassortment potential observed in the minigenome assays, we attempted to generate all reassortants via reverse genetics. To do this, we first constructed a rBATV by amplifying the full-length S, M, and L genome segments of BATV strain MM2222 from infected Vero E6 cells. Reverse transcription was performed using random primers and segment-specific oligonucleotides designed from complete GenBank sequences (S: JX846595; M: JX846596; L: JX846597; Table 1). Full-length cDNAs were cloned into the TVT7R(0,0) plasmid for transcription by T7 RNA polymerase. rBATV was rescued by transfecting BSR-T7/5 cells with pT7BATVS, pT7BATVM, and pT7BATVL plasmids. Cytopathic effects (CPE) were evident 5–7 days post-transfection (d.p.t.), and plaque assays confirmed successful virus recovery.

**Table 1:** Oligonucleotides used in this study.

Virus	Segment	Fwd/Rev/Probe	Sequence (5’- 3’)
rBATV	S (full length)	Fwd	AGTAGTGTACTCCACACT
		Rev	AGTAGTGTGCTCCACCTA
	M (full length)	Fwd	AGTAGTGTACTACCGATA
		Rev	AGTAGTGTGCTACCGATA
	L (amplicon 1)	Fwd	AGTAGTGTACTCCTATATAAAGA
		Rev	GGCTCACTGATCATTTCATCG
	L (amplicon 2)	Fwd	CGACAGACTTTACGAATTATTA
		Rev	GTCTGGATCGTTTCTCTCTGCTT
	L (amplicon 3)	Fwd	CATGATATACCGGTAGAACTGAA
		Rev	AGTAGTGTGCTCCTATATAAAAG
rBUNV	S	Fwd	CAATTTCTCTGGTTCACTGACTTTC
		Rev	TCAGTGGATTCCTTGCCAGGTACC
		Probe (FAM)	CAGTTCCTGACGATGGTCTTAC
rBATV	S	Fwd	TCCACCCAAGTCCTGATACT
		Rev	AAGACCATGCGCCAAAGA
		Probe (FAM)	TGGGCTTGGAAGCATCAACTTGGA

**Fig 2:**
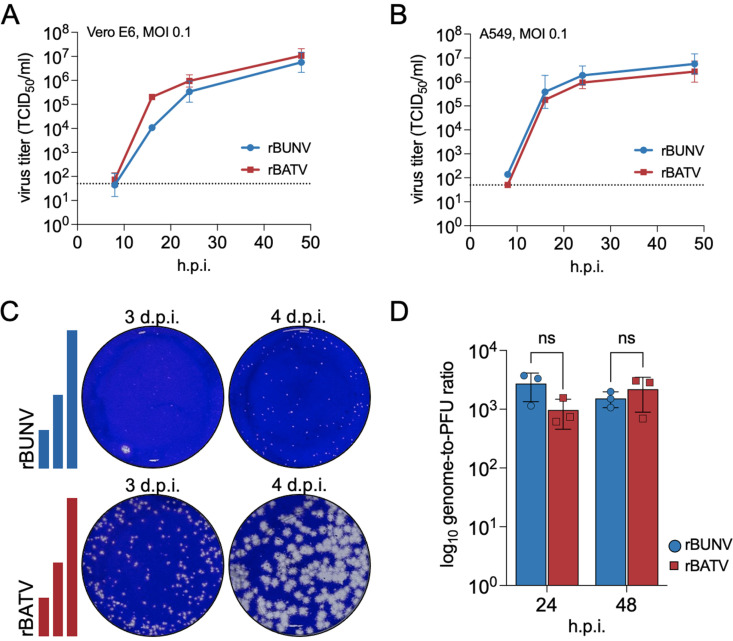
Rescue and characterization of rBATV. (A and B) Viral growth curves in Vero E6 (A) and A549 (B) cells infected with either rBUNV or rBATV at MOI 0.1. Infectious viral titers were determined at 8, 18, 24, and 48 h.p.i. and represented as TCID_50_/ml. Error bars represent standard deviations (n = 3), and the dotted line represents the limit of detection (LoD). (C) Viral plaque phenotype comparison between rBUNV and rBATV. Plaque assays in Vero E6 cells were fixed in 4% PFA and stained with crystal violet. Representative images are shown out of an n = 3. (D) Genome copies-to-PFU ratio of rBUNV and rBATV in Vero E6 cells at 24 and 48 h.p.i. RNA was extracted from cell-free supernatant virus stock, and genome copies determined by RT-qPCR. Virus stock titers were determined by plaque assay. Error bars represent standard deviation (n = 3).

Prior to generating reassortants, we compared the *in vitro* characteristics of rBATV and rBUNV and found that both viruses exhibited similar replication kinetics in IFN-deficient Vero E6 and IFN-competent A549 cells (Fig 2A and B). However, rBATV consistently formed larger plaques in Vero E6 cells by 3 days post-infection (d.p.i.) compared to rBUNV, which required an additional day to produce visible plaques (Fig 2C). Despite these plaque size differences, both viruses caused similar levels of CPE by 3 d.p.i. and their genome-to-PFU ratios were also comparable around 24- and 48-hours post-infection (h.p.i.) (Fig 2D).

**Fig 3:**
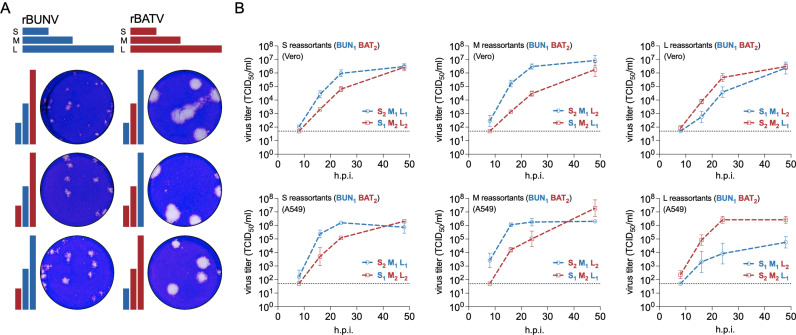
Rescue and characterization of rBUNV-rBATV reassortants. (A) Viral plaque phenotype comparison between reassortants. Plaque assays in Vero E6 cells were fixed at 4 d.p.i. in 4% PFA and stained with crystal violet. Representative images are shown out of an n = 2. (B) Viral growth curves in Vero E6 (A) and A549 (B) cells infected with various reassortants at MOI 0.1. Infectious viral titers were determined at 8, 18, 24, and 48 h.p.i. and represented as TCID_50_/ml. Error bars represent standard deviations (n = 3), and the dotted line represents the LoD.

Next, we swapped the S, M, and L segments between rBUNV_1_ and rBATV_2_ and successfully generated all six unique reassortants, including an NRIV-like virus (BATV M + BUNV S/L, i.e., S_1_M_2_L_1_). We then analyzed all reassortants for viral fitness, revealing that those containing the rBATV M (M_2_) segment consistently formed larger plaques than those with the rBUNV M (M_1_) segment, indicating a dominant role of the M segment in influencing viral spread (Fig 3A). Regarding viral replication, the only segment of rBUNV_1_ and rBATV_2_ consistently represented in the faster-growing reassortants of all pairings was the rBATV_2_ S segment (S_2_) (Fig 3B). The M and L reassortants demonstrated that rBUNV_1_ acted deterministically as the heterologous segment, as shown by both S_2_M_1_L_2_ and S_2_M_2_L_1_ exhibiting faster growth than their M and L counterparts (including NRIV-like virus), respectively. The only reassortant failing to reach the same endpoint titer as its counterpart was S_1_M_1_L_2_, exhibiting nearly two logs lower viral titers in A549 cells at 48h.p.i. The S segment reassortants, S_2_M_1_L_1_ and S_1_M_2_L_2_, displayed similar growth rates in both cell types. These findings highlight the segment-specific contributions to viral replication and plaque morphology, emphasizing the potential implications for viral fitness among the reassortants.

### A multiplex HCR RNA-FISH assay enables single-molecule detection and quantification of rBUNV and rBATV genomic segments

**Fig 4:**
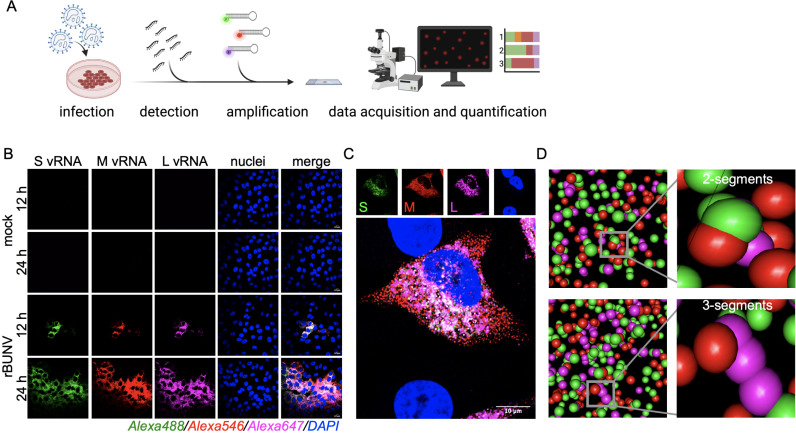
Development of a bunyavirus HCR RNA-FISH assay. (A) Schematic of the HCR assay workflow (Created in BioRender. Tilston, N. (2025) https://BioRender.com/9ry0hhq). (B-C) Vero E6 cells were either mock-infected or infected with rBUNV at an MOI 0.1 and fixed at defined time points. The S (green), M (red), and L (magenta) genome segments were detected using HCR RNA-FISH. Nuclei (blue) were visualized by DAPI staining. Representative images are shown out of an n = 3. Scale bars 10 μm (D) Three-dimensional representation illustrating the intracellular spatial distribution of genome segments.

To investigate the intracellular distribution and co-localization of the S, M, and L genome segments during infection, we developed and validated a multiplex single-molecule HCR RNA-FISH assay. This assay employs DNA probes that specifically hybridize to target RNA sequences, triggering a hybridization chain reaction (HCR) with hairpin DNA amplifiers. The amplifiers remain inactive until bound to the probe-target complex, which initiates a cascade that generates fluorescently labeled polymers. This non-enzymatic amplification process enables precise detection and visualization of individual vRNA segments within infected cells [19,20] (Fig 4A).

Using this assay, we detected the S, M, and L RNA segments of rBUNV at 12- and 24-h in Vero E6 cells, with no detectable signals in mock-infected controls, validating the specificity of the assay for vRNA detection (Fig 4B). Signal intensity and segment abundance appeared widely dispersed at 24 h compared to 12 h, consistent with ongoing replication and accumulation of vRNA over time. To further analyze the spatial organization, we used confocal microscopy to acquire Z-stack images and reconstruct the intracellular localization of vRNA segments in three dimensions. These reconstructions revealed spatially distinct yet overlapping distributions of the S, M, and L segments within the cytoplasm of infected cells, suggesting possible co-localization. We also built a custom CellProfiler pipeline to integrate these images to quantitatively analyze segment distribution and co-localization patterns (Fig 4C and D).

**Fig 5:**
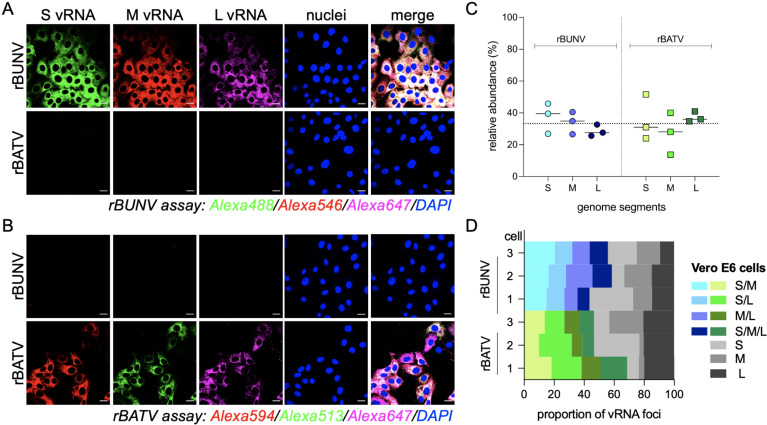
Validation of the bunyavirus HCR RNA-FISH Assay. (A-B) Vero E6 cells were infected with rBUNV or rBATV at an MOI of 1 and fixed 8 h.p.i. The S, M, and L genome segments were visualized using the HCR RNA-FISH assay. Representative images are shown out of an n = 2. Scale bars 10 μm. (C) Quantitative analysis of relative abundance for S, M, and L genome segments in Vero E6 cells infected with rBUNV and rBATV. Each data point represents the percentage of cells with detectable fluorescence for a specific genome segment. (D) Proportion of vRNA foci within each cell (Vero E6) co-localizing two or more genome segments.

Specificity tests demonstrated no cross-reactivity between the rBUNV and rBATV probe sets, as each assay successfully detected its corresponding vRNA without interference (Fig 5A and B). At 24 h.p.i., the relative abundance of the S, M, and L genome segments in Vero E6 cells was comparable for rBUNV and rBATV, though rBUNV exhibited a slight gradient in abundance (S > M > L) (Fig 5C). In both rBUNV and rBATV-infected cells, S vRNA, M vRNA, and L vRNA appeared to be widely distributed throughout the cytoplasm, and co-localization analysis revealed that while the segments were frequently found together, there were distinct regions where segments localized independently. Quantitative co-localization analysis indicated that both rBUNV and rBATV formed two-segment and three-segment vRNA foci, though the frequency and distribution varied (Fig 5D). The HCR RNA-FISH assay highlighted the robust replication and widespread cytoplasmic presence of all three genome segments in both viruses.

### Differential pathogenicity of rBUNV and rBATV in IFNAR^-/-^ mice

**Fig 6:**
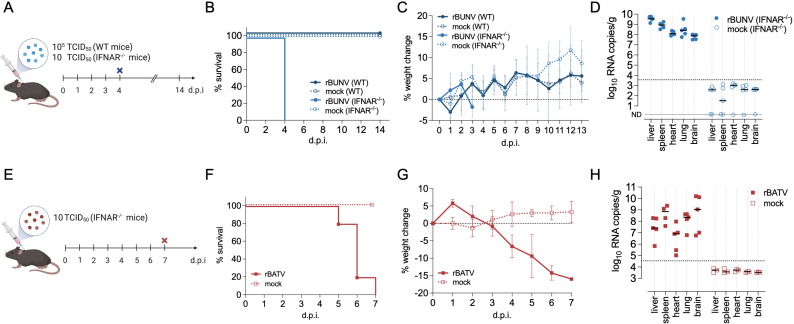
rBUNV and rBATV infection in mice. (A and E) Experimental outline of mice infection (Created in BioRender. Tilston, N. (2025) https://BioRender.com/9ry0hhq). Mice were SC infected with either rBUNV, rBATV, or Opti-MEM (B and F). Survival curves of infected mice (n = 5; A-D and n = 3 for mock only in E-H) (C and G). Percent weight change of mice from baseline during the course of infection. (D and H) Viral load per gram of tissue in the liver, spleen, lung, heart, and brain of mock, rBUNV or rBATV infected mice as measured by RT-qPCR (QuantStudio 5 Applied Biosystems). The dashed line represents the LoD and ND signifies non-detected.

To compare the pathogenicity of rBUNV and rBATV, we performed infection studies in mice (Fig 6A and E). Immunocompetent C57BL/6 mice subcutaneously (SC) inoculated at the scruff with 10^5^ TCID_50_ of rBUNV showed no mortality or significant weight loss throughout the study period, maintaining stable health parameters (Fig 6B and C). In contrast, IFNAR^-/-^ mice infected with as little as 10 TCID_50_ of rBUNV experienced 100% mortality by 4 d.p.i., preceded by significant weight loss, nasal bleeding, hunched posture, and squinted eyes. High vRNA loads were detected in the liver, spleen, lung, heart, and brain, suggesting a systemic infection (Fig 6D). These results highlight that while rBUNV is non-lethal in immunocompetent mice, it causes severe systemic lethal disease in the absence of type I IFN signaling, underscoring the critical role of the host immune response in controlling infection.

In IFNAR^-/-^ mice at a 10 TCID_50_ dose, rBATV caused delayed mortality compared to rBUNV, with death occurring between 5 and 7 d.p.i. (Fig 6F). Weight loss progressed more gradually during rBATV infection but became pronounced by 5 d.p.i., coinciding with the onset of mortality (Fig 6G). Unlike rBUNV-infected mice, rBATV-infected animals did not exhibit external bleeding. However, some mice displayed a hunched posture, and splenomegaly was noted in a few cases. Analysis of vRNA levels revealed considerable variability across tissues, with a trend toward higher vRNA levels in the brain than in the liver (Fig 6H). This contrasts with rBUNV infection, where viral loads are predominantly highest in the liver (Fig 6D). These findings highlight distinct pathogenic profiles for rBUNV and rBATV in IFNAR^-/-^ mice. While rBUNV leads to rapid disease progression, severe liver pathology, and mortality by day 4, rBATV follows a slower disease course with 100% mortality by day 7.

### M-segment reassortants induce a comparable disease pathology in IFNAR^-/-^ mice

**Fig 7:**
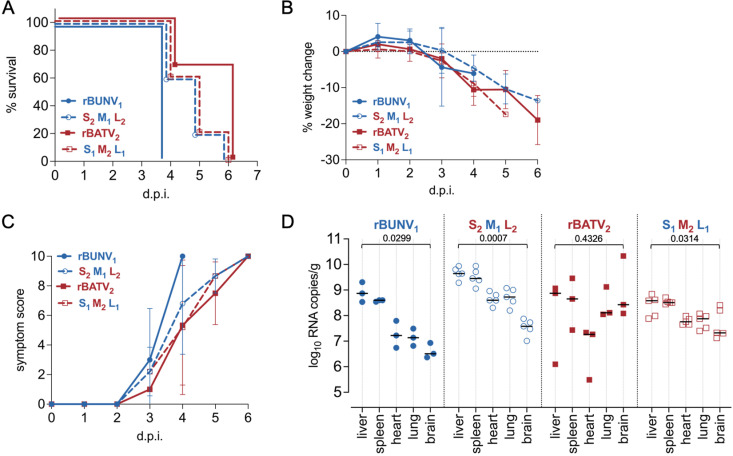
rBUNV-rBATV reassortant infection in mice. (A) Survival curves of mice infected with rBUNV_1_ (n = 3), rBATV_2_ (n = 3), S_2_M_1_L_2_ (n = 5) or S_1_M_2_L_1_ (n = 5) (B) Percent weight change of mice from baseline during the course of infection. (C) Symptom score for all infected mice (D) Viral load per gram of tissue in the liver, spleen, heart, lung, and brain of all infected mice as measured by RT-qPCR (QuantStudio 5 Applied Biosystems). Comparisons between the liver and brain were performed for each virus group using a one-way ANOVA.

As NRIV is an M-segment reassortant and most orthobunyavirus reassortants found in nature involve the M segment, we focused our initial study on M-segment reassortants. Specifically, we analyzed an NRIV-like virus (rBATV_2_ M + rBUNV_1_ S/L, i.e., S_1_M_2_L_1_) and its counterpart (rBUNV_1_ M + rBATV_2_ S/L, i.e., S_2_M_1_L_2_) alongside the parental viruses, rBUNV_1_ and rBATV_2_. To assess M segment reassortment on pathogenicity, IFNAR^-/-^ mice were subcutaneously infected with 10 TCID_50_ of rBUNV, rBATV, or their reassortants (S_1_M_2_L_1_ and S_2_M_1_L_2_). Survival analysis showed that rBUNV was lethal by 4 d.p.i., whereas rBATV and the reassortants caused mortality between 5 and 6 d.p.i., with the reassortants exhibiting a staggered survival profile (Fig 7A). All virus-infected groups showed progressive weight loss (Fig 7B), with rBUNV-infected mice exhibiting the steepest decline by day 3, consistent with its earlier lethality. The reassortants displayed weight loss patterns similar to rBATV. Symptom scores rose sharply in rBUNV-infected mice by day 3, while rBATV and the reassortants demonstrated a more gradual increase, indicating delayed disease progression (Fig 7C). Across all infected groups, mice displayed hunched posture and ruffled fur. Notably, one rBUNV M + rBATV S/L (S_2_M_1_L_2_) reassortant mouse developed white eye discharge, and recessed eyes were observed in some rBATV- and rBATV M + rBUNV S/L (S_1_M_2_L_1_)-infected mice.

Tissue-specific vRNA loads at the experimental endpoint revealed that rBUNV and its M segment reassortant (rBUNV M + rBATV S/L, i.e., S_2_M_1_L_2_) exhibited consistently high viral loads in the liver and spleen (Fig 7D). In contrast, rBATV displayed a more heterogeneous distribution of vRNA across tissues, with greater variability in vRNA levels between individual animals. Its M segment reassortant (rBATV M + rBUNV S/L, i.e., S_1_M_2_L_1_) exhibited a similar pattern of tissue dispersion, though reduced variability between animals. These observations suggest that while the M segment may contribute to tissue tropism, additional segment interactions or virus-specific factors likely influence viral dissemination.

### rBUNV, rBATV, and reassortants induce severe liver pathology in IFNAR^-/-^ mice

**Fig 8:**
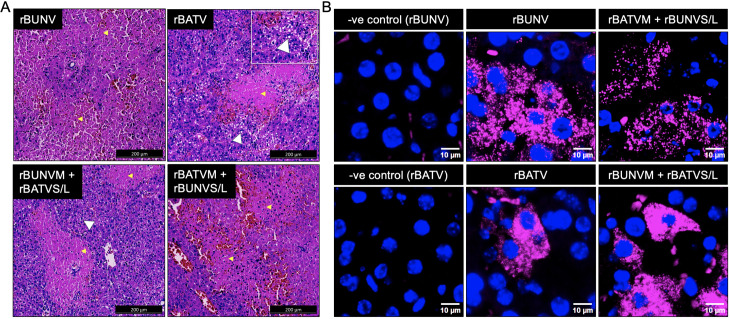
Liver pathology in mice infected with rBUNV, rBATV, and reassortants. (A) Representative images of liver sections showing severe multifocal hepatic necrosis in mice infected with rBUNV, rBUNV M + rBATV S/L (S_2_M_1_L_2_), rBATV, or rBATV M + rBUNV S/L (S_1_M_2_L_1_), as indicated by yellow arrows. Areas of fatty acid accumulation are highlighted with white arrows. A magnified view of fatty acid accumulation in rBATV-infected liver is shown within the image. Sections were stained with H&E, visualized under light-field microscopy, and imaged using an Aperio whole-slide imaging system. Scale bars, 200 μm. (B) Representative images of liver tissue sections showing L segment vRNA (magenta) detected using HCR RNA-FISH. Nuclei are visualized with Hoechst staining (blue). Scale bars 10 μm. -ve control (rBUNV), rBUNV, rBATVM + rBUNVS/L were stained using BUNV HCR assay. -ve control (rBATV), rBATV, rBUNVM + rBATVS/L were stained using BATV HCR assay.

Liver and brain tissues from infected IFNAR^-/-^ mice were examined for histopathological signs of disease using hematoxylin and eosin (H&E)-stained sections. Brain sections revealed no abnormalities across all groups. In contrast, liver sections showed extensive hepatic necrosis and hemorrhage in all infection groups (Fig 8A; yellow arrows), with comparable severity among mice infected with rBUNV, rBATV, or their M-segment reassortants. Notably, fatty liver changes (indicative of hepatic steatosis) were exclusively observed in rBATV-infected mice and, to a lesser extent, in mice infected with the reassortant rBUNV M + rBATV S/L (i.e., S_2_M_1_L_2_; Fig 8A; white arrows). To investigate viral replication in the liver, HCR RNA-FISH assays were performed on the formalin-fixed sections using probes targeting the L segment. Here, we used probes targeting the L segment alone to simplify detection. Our results revealed extensive levels of rBUNV L vRNA in the livers of infected mice, with relatively lower levels of rBATV L vRNA detected in rBATV-infected animals (Fig 8B).

**Fig 9:**
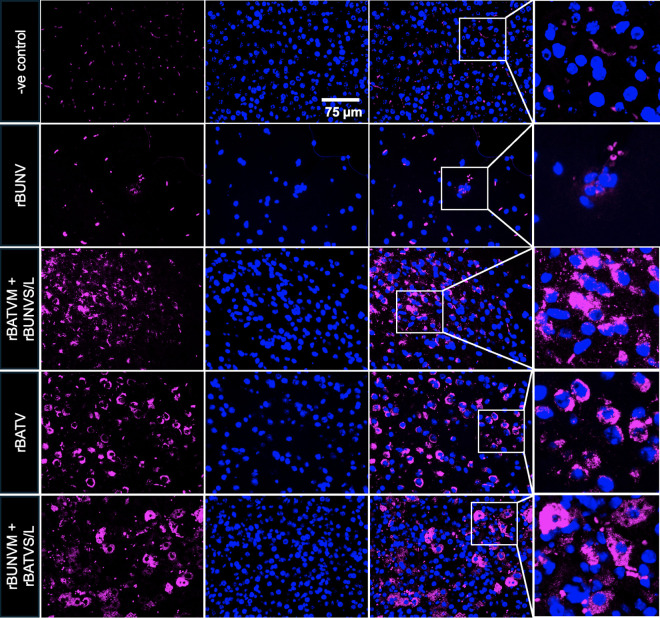
Detection of vRNA in the brain of IFNAR^-/-^ mice infected with rBUNV, rBATV, and reassortants using HCR RNA-FISH. Representative images of brain tissue sections showing L segment vRNA (magenta) detected using HCR RNA-FISH. Nuclei are visualized with Hoechst staining (blue). rBUNV, rBATVM + rBUNVS/L (S_1_M_2_L_1_), were stained using BUNV HCR assay, while -ve control, rBATV, and rBUNVM + rBATVS/L (S_2_M_1_L_2_), were stained using BATV HCR assay.

Although brain sections showed no overt pathological changes, HCR RNA-FISH assays revealed significant differences in vRNA levels among infection groups. High levels of L vRNA were detected in the brains of rBATV-infected mice and both M-segment reassortants (Fig 9). In contrast, rBUNV-infected mice displayed sparse and isolated pockets of vRNA within the brain tissues, indicating limited neuroinvasion.

These findings confirm that rBUNV, rBATV, and their M-segment reassortants replicate efficiently in the liver of IFNAR^-/-^ mice, with all tested viruses inducing severe liver pathology. Notably, rBATV and the reassortant rBUNV M + rBATV S/L (S_2_M_1_L_2_) uniquely induced hepatic steatosis. Furthermore, the marked differences in replication in the brain between rBUNV and rBATV suggest potentially distinct tropism and pathogenic mechanisms among these viruses.

## Discussion

This study provides critical insights into the reassortment and pathogenicity of rBUNV and rBATV, demonstrating the viability of all six possible reassortant combinations *in vitro* and highlighting segment-specific contributions to viral replication and disease outcomes. By integrating advanced molecular tools, such as the multiplex single-molecule HCR RNA-FISH assay [20], we visualized vRNA segments at the single-molecule level, revealing their intracellular distribution during infection. The HCR RNA-FISH assay developed in this study represents a powerful tool for investigating segment-specific interactions. Future studies will expand on this approach to reconstruct high-resolution three-dimensional images, enabling a deeper exploration of the co-localization dynamics for the S, M, and L genome segments during rBUNV and rBATV co-infections.

This work builds on our previous studies utilizing minigenome assays to investigate reassortment potential [17]. These assays provide a valuable platform for studying viral replication and transcription independently of infectious virus. Minigenomes, composed of viral UTRs flanking a reporter gene in a negative-sense orientation, allow precise assessment of whether N and RdRp proteins can recognize and utilize UTRs as functional promoters. Our minigenome results for BUNV and BATV indicated that reassortment should, in principle, be feasible across all six combinations. However, only one reassortant, NRIV (BATV M + BUNV S/L), has been observed in nature. Recognizing the inherent limitations of minigenome assays, such as their inability to fully capture the complexity of viral infection and reassortment, we used reverse genetics to generate all six reassortants. This approach not only confirmed their viability but also validated the findings of the minigenome assays, further underscoring their value, particularly when studying high-containment pathogens. Our results suggest that viral fitness, not simply compatibility, may ultimately determine the successful viability of specific reassortants during natural co-infection.

Our findings also reveal distinct pathogenic profiles for rBUNV, rBATV, and their reassortants in IFNAR^-/-^ mice. While rBUNV was lethal by 4 d.p.i., with systemic infection, hemorrhage, high vRNA loads, and severe liver necrosis, rBATV induced a slightly delayed response with additional pathologies, such as hepatic steatosis, highlighting potentially unique pathogenic mechanisms associated with this virus. The M segment reassortants tested exhibited intermediate phenotypes, further emphasizing the segment-specific contributions to disease severity. The implications of this work are significant for understanding how reassortment drives the emergence of strains with altered pathogenicity and fitness. Future research will investigate the role of the M segment in immune modulation and host responses, as well as the interactions between reassortant viruses and host factors, to uncover the molecular underpinnings of pathogenesis. Extending this framework to vector studies could elucidate how reassortment occurs in natural transmission cycles, further advancing our understanding of viral evolution and emergence. A significant outcome of our study is the development of an integrated approach combining advanced imaging techniques with classical virology methods. This will enable comprehensive investigations of vRNA behavior and segment-specific contributions to reassortment. The HCR RNA-FISH assay offers a highly sensitive method to study the spatial dynamics of vRNA segments in greater detail.

In conclusion, this study establishes a robust framework for characterizing orthobunyavirus reassortment and elucidating segment-specific contributions to viral fitness, replication, and pathogenesis. By combining innovative molecular tools with *in vitro* and *in vivo* methods, this work provides a strong foundation for future investigations into the evolutionary dynamics of orthobunyaviruses and their implications for public health.

## Materials and methods

### Ethics statement

All animal studies were conducted in accordance with protocols approved by the Indiana University School of Medicine (IUSM) Institutional Animal Care and Use Committee (IACUC; protocol #22080). Experiments involving rBUNV and rBATV individually were performed in Biosafety Level 2 (BSL-2) or Animal BSL-2 (ABSL-2) facilities, while experiments comparing rBUNV, rBATV, and their reassortants were conducted in IUSM’s BSL-3 and ABSL-3 facilities.

### Viruses and plasmids

Vero E6 cells (African green monkey kidney cells), BSR-T7/5 cells (baby hamster kidney cells), which stably express T7 RNA polymerase [21] maintained with 1 mg/ml of G418 (Geneticin; Invitrogen) and A549 (human alveolar adenocarcinoma epithelial cells) were grown in Dulbecco’s modified Eagle medium (DMEM; Gibco) supplemented with 2 – 10% (V/V) fetal bovine serum (Gibco). All cells were grown at 37°C and 5% CO_2_.

Rescue plasmids for generating rBUNV (pT7riboBUNS, pT7riboBUNM, and pT7riboBUNL), along with the expression plasmids pTM1-BUNV-N and pTM1-BUNV-L, have previously been described [18]. Rescue plasmids to generate rBATV were constructed by amplifying the positive-sense cDNA of the S, M, and L segments (Table 1). These cDNA amplicons were then assembled via Gibson Assembly (NEBuilder HiFi DNA assembly, NEB) into plasmids between a T7 promoter and hepatitis delta ribozyme, followed by a T7 terminator [TVT7R(0,0)] [22], generating pT7BATVS, pT7BATVM, and pT7BATVL plasmids. Expression plasmids encoding the N and RdRp proteins were generated by PCR amplifying the N and RdRp coding regions from pT7BATVS and pT7BATVL, respectively. The amplicons were then ligated into the eukaryotic expression vector pCG using *Asc* I and *Spe* I restriction sites to generate pCG-BATV-N and pCG-BATV-RdRp. Minigenome plasmids for the S, M, and L segments of BUNV and BATV were engineered using synthetic gene fragments (gBlocks, IDT) containing red fluorescent protein (dTomato; dTom), blue fluorescent protein (TagBFP), and green fluorescent protein (Venus) flanked on either side by the S, M and L segment UTRs, respectively. Unlike the rescue plasmids, all minigenomes were cloned into the negative-sense orientation into TVT7R(0,0), generating pT7(-)BUNVSdTom, pT7(-)BUNVMTagBFP, and pT7(-)BUNVLVenus for BUNV and pT7(-)BATVSdTom, pT7(-)BATVMTagBFP, and pT7(-)BATVLVenus for BATV. All plasmids described here were sequence verified using Sanger sequencing (Genewiz, USA).

Viruses rBUNV, rBATV, and the six reassortants were rescued in BSR-T7/5 cells as previously described [23]. Successful virus rescue was confirmed by observing CPE by phase-contrast microscopy and plaque assays. Virus stocks were grown and titrated in Vero E6 cells using a 50% tissue culture infectious dose assay (TCID_50_).

### Minigenome assay

Sub-confluent monolayers of BSR-T7/5 cells grown in 24-well plates were transfected with 250 ng of minigenome plasmids, pT7(-)BUNVSdTom, pT7(-)BUNVMTagBFP, pT7(-)BUNVLVenus, pT7(-)BATVSdTom, pT7(-)BATVMTagBFP, or pT7(-)BATVLVenus. Expression plasmids for BUNV or BATV N and RdRp proteins were co-transfected at 125 ng per well. Transfections were performed using Lipofectamine 2000 (ThermoFisher) and at 2 days post-transfection (d.p.t.), cells were visualized for fluorescence to assess reporter gene expression.

### TCID_50_ and plaque assays

TCID_50_ assays were performed in Vero E6 cells at a density 10^4^ cells per well in 96-well plates and infected with a 10-fold serial dilution of virus. At 7 d.p.i. CPE was recorded, and viral titers expressed as tissue culture infectious dose (TCID_50_ units). Plaque assays were performed in 6-well plates with Vero E6 cells at a density of 10^5^ cells/ml. Cell monolayers were infected with a 200 μl inoculum of virus diluted in Opti-MEM. Cells were then overlaid with 0.6% Avicel (FMC, Avicel RC-591) in 2X minimum essential medium (MEM)/2% FBS. Cells were fixed at 3 d.p.i. with 4% (w/v) paraformaldehyde (PFA) in PBS for 30 mins and plaques visualized using crystal violet.

### Viral growth kinetics

Vero E6 and A549 cells (2.0 x 10^5^ cells/ml, 24-well plate) were infected with either rBUNV, rBATV, or their reassortants at a multiplicity of infection (MOI) 0.1 for 1 h at 37°C. Cell monolayers were washed 3X with D-PBS (Gibco), and then provided with 2% FBS DMEM. At the desired time points, supernatant was collected, and infectious viral titers were determined by TCID_50_ assay.

### Quantitative RT-qPCR

rBUNV and rBATV vRNA were quantified using primers targeting the S genome segment (Table 1). The working concentration for the primers was 10 μM for the probe 5 μM. To generate RNA for a standard curve, pT7riboBUNS and pT7BATVS plasmids containing the entire S segment of rBUNV and rBATV, respectively, were linearized via restriction enzyme digest, purified, and used as a template for *in vitro* RNA transcription (MEGAscript T7 transcription kit; Invitrogen). Resulting RNA was diluted to known copies per ml in RNase-free water and serially diluted for each assay. RT-qPCR was performed using the Luna Universal Probe One-Step RT-qPCR Kit (NEB). The assay was performed on the QuantStudio 5 (ThermoFisher Scientific) using the following conditions: 55°C for 10 minutes, 95°C for 1 minute, and then 40 cycles of 95°C for 10 seconds and 60°C for 1 min. Genome-to-PFU (i.e., plaque-forming units) ratios were determined by performing plaque assays and quantifying genome copies from the same viral stock. For the animal studies, RNA levels were reported as log vRNA copies per gram of tissue, with the limit of detection (LoD) calculated based on the highest cycle threshold (CT) value detected in the standard curve for each assay. To measure vRNA levels for the reassortants, rBUNV M + rBATV S/L and rBATV M + rBUNV S/L, we used rBATV S primers and rBUNV S primers, respectively.

### Hybridization chain reaction (HCR) RNA-FISH assay

Probe sets targeting the S, M, and L segments of each virus were designed using the DIY probe generator pipeline [24]. All probes were screened for internal specificity (i.e., cross-reactivity with other segments of the same virus) as well as against host cell transcripts to minimize background. 

The HCR assay was performed following protocols published by Molecular Instruments, Inc. To visualize rBUNV and rBATV genome segments in Vero E6 cells, cells were seeded in 24-well plates containing coverslips at a density of 10^5^ cells per well. Infected cells were fixed using 4% paraformaldehyde (PFA) for 30 minutes at room temperature (RT). Fixed cells were permeabilized overnight at -20°C in 70% ethanol. The next day, samples were air-dried and washed twice with 2X Saline-sodium citrate (SSC) buffer (300 µL, 5 minutes each). Cells were pre-hybridized in probe hybridization buffer (Molecular Instruments) for 30 minutes at 37°C before adding a probe solution, prepared with 1.2–3 µL of each probe set (1 µM stock) specific for BUNV or BATV S, M, and L segments in Tris-EDTA (TE) buffer (S1–S2 Tables; purchased from IDT as oligopools). Incubation with the probe solution was conducted overnight at 37°C. After probe hybridization, the samples were washed 4X with probe wash buffer (Molecular Instruments) at 37°C, followed by two washes with 5X SSCT (SSC + 0.1% Tween 20) at room temperature. Amplification buffer (Molecular Instruments) was added for pre-amplification (30 minutes, RT), and a prepared hairpin solution (300 µL per sample) was added (rBUNV S = B1-488, M = B2-546, L = B3-647 and rBATV S = B4-594, M = B5-514 and L = B2-647 purchased from Molecular Instruments). The samples were incubated overnight at RT in the dark. Cells were then washed 5X with 5X SSCT, stained with DAPI or Hoechst for 30 minutes, and mounted using an antifade solution. Slides were imaged on an Invitrogen EVOS fluorescent microscope M5000 or a Zeiss LSM 880 Airyscan.

### Visualization and colocalization analysis of vRNA

The acquired confocal four-color Z-stacks were split into individual channels using FIJI (ImageJ2, V2.3.0) [25]. The Radial Symmetry-FISH (RS-FISH) plugin was used to identify spots for all vRNA signals [26]. The corresponding output RS-files were imported into R and projected into 3-dimensional space for visualization. Deconvolution was performed in ZEN Blue 3.11(Zeiss) to quantify the co-localization of vRNA. Background subtraction was performed on all channels in FIJI (ImageJ2, V2.3.0). CellProfiler analysis: a custom CellProfiler [27] pipeline was used to identify objects (vRNA) and calculate the overlap of two and three segments in each slice. The total number of single, double, and triple vRNA objects were analyzed in Microsoft Excel and plotted in GraphPad Prism 9.

### Animal study design

Six-week-old female C57BL/6 wild-type (WT) or IFNAR^-/-^ mice (Jackson Laboratory) were housed in HEPA filtration cages in either the ABSL-2 facility or the ABSL-3 facility, with ad lib access to food and water. WT mice were SC infected with 10^5^ TCID_50_ of rBUNV, while IFNAR^-/-^ mice were infected with 10 TCID_50_ of rBUNV, rBATV or the reassortants (rBATV S/L + rBUNVM and rBUNV S/L + rBATV M) diluted in Opti-MEM to a volume of 100 μl. Control animals were inoculated with only Opti-MEM in the same volume. Mice were monitored daily for clinical signs of disease, and weights were recorded. Mice were euthanized according to a predetermined clinical scoring method as previously described [22]. At the time of euthanasia, mice were anesthetized with isoflurane and terminally exsanguinated for serum collection. Following a cervical dislocation, the liver, spleen, heart, lungs, and brain were collected for viral isolation and RNA extraction, followed by RT-qPCR for vRNA analysis. Whole organs were also collected for histological analysis and vRNA detection. Here, tissues were fixed in 4% PFA, paraffin-embedded, and cut by microtomy (5 μM sections). Tissue sections were mounted on glass slides and either stained with H&E or subjected to an HCR assay. For viral load detection, tissues were collected in pre-weighed tubes containing 500 µl of sterile PBS with 2X Antibiotic-Antimycotic (Gibco) and weighed before homogenization using a Bead Mill 24 Homogenizer (Fisherbrand). 100 µl of homogenized tissue was added to 400 µl of TRIzol LS regent (Ambion) and purified using the Direct-zol RNA purification kit (Zymo Research).

### Statistical analysis

RT-qPCR data were analyzed in Microsoft Excel and plotted in GraphPad Prism 9. Genome-to-PFU ratios (Fig 2) were compared using a two-way ANOVA with Sidak’s multiple comparison test in GraphPad Prism 9. RNA copy numbers per gram in the liver versus brain tissue (Fig 7) were compared using a one-way ANOVA.

## Supporting information

S1 Table rBUNV HCR probes used in the study (Figs 4, 5, 8 and 9). (DOCX)

S2 Table rBATV HCR probes used in the study (Figs 5, 8 and 9). (DOCX)
