# Supplementary material for: Probing orthobunyavirus reassortment using Bunyamwera and Batai viruses as models
Source: PLoS Negl Trop Dis. 2025 May 30;19(5):e0013120. doi: 10.1371/journal.pntd.0013120 (PMC12169594; doi:10.1371/journal.pntd.0013120)
Supplement: S1 Table — (DOCX) [file pntd.0013120.s001.docx]

**Supplementary Table 1: rBUNV HCR probes used in the study (Figures 4, 5, 8 and 9).**

| **Segment** | **OligoPool name** | **Sequence** |
| --- | --- | --- |
| S | B1_BUNVS_9_Dla80 | GAGGAGGGCAGCAAACGGaaTGACCACATACGAATCTTCTACATT |
|  |  | GCGTGTCCACACCACTGGGCTTAGTtaGAAGAGTCTTCCTTTACG |
|  |  | GAGGAGGGCAGCAAACGGaaAAAAGAAGTGAATGGGAAGTTACAC |
|  |  | GGACGCGAGATTAAAACTAGTCTCGtaGAAGAGTCTTCCTTTACG |
|  |  | GAGGAGGGCAGCAAACGGaaATAATACGAATTTTCCTGGCAACCG |
|  |  | ACCTTGGGGGCTGGAAGATTACTGTtaGAAGAGTCTTCCTTTACG |
|  |  | GAGGAGGGCAGCAAACGGaaAAAAGAATGGGATCACTTGGAATGA |
|  |  | AATCAAAAATAATCAACCCTTTGGCtaGAAGAGTCTTCCTTTACG |
|  |  | GAGGAGGGCAGCAAACGGaaAGGATCAGAGATGTTCTTAGGAACT |
|  |  | AGAGGAAGTTTATCTCTCTTTCTTCtaGAAGAGTCTTCCTTTACG |
|  |  | GAGGAGGGCAGCAAACGGaaTACAAAGTTCAGCGCAAGGAAATGG |
|  |  | AGATTCTACCCCTTAGCAATCGGGAtaGAAGAGTCTTCCTTTACG |
|  |  | GAGGAGGGCAGCAAACGGaaAGAGGTACATGGGACTAGAAGCAGC |
|  |  | CAAAATACCTTGAGAAAACAATGCGtaGAAGAGTCTTCCTTTACG |
|  |  | GAGGAGGGCAGCAAACGGaaTCAGTCTGCACTGACAGTTGTCTCT |
|  |  | TTGGACTGTTAGTAAATTGACAGAAtaGAAGAGTCTTCCTTTACG |
|  |  | GAGGAGGGCAGCAAACGGaaGCAGCTGCCAGGGACTTCCTTGCTA |
|  |  | TTAGGTTGGAAGAAAACCAATGTTAtaGAAGAGTCTTCCTTTACG |
| M | B2_BUNVM_26_Dla50 | CCTCGTAAATCCTCATCAaaGCCCAAATTATATTTCAGACAGACA |
|  |  | TGCACTATAACAGTTGATAAGGAAAaaATCATCCAGTAAACCGCC |
|  |  | CCTCGTAAATCCTCATCAaaCTATCAGCACTGGCTGGTTTAAGTC |
|  |  | TTAATCATTTTGAAGTGACAGGAACaaATCATCCAGTAAACCGCC |
|  |  | CCTCGTAAATCCTCATCAaaATCCCAGTGTTTATGCCCATAGCTT |
|  |  | ACAAAAACATACATTTGCTACGTGTaaATCATCCAGTAAACCGCC |
|  |  | CCTCGTAAATCCTCATCAaaGTAAGAAATGCACTTGTTGCGGATT |
|  |  | CTTATGGATGGGCGTACAACAGGAGaaATCATCCAGTAAACCGCC |
|  |  | CCTCGTAAATCCTCATCAaaCTTTGTAACACCCATAGAAGGGACT |
|  |  | CATCTTACTCTCAGTGCTGATTCTAaaATCATCCAGTAAACCGCC |
|  |  | CCTCGTAAATCCTCATCAaaACTTTAGATGAGATAGCAGATGTCC |
|  |  | ACAAACTACCCTACTGATCAGAAATaaATCATCCAGTAAACCGCC |
|  |  | CCTCGTAAATCCTCATCAaaTGCAGGAGTAGCATTGGGGTTAACA |
|  |  | CTTCGGTGCAGGTCTGACCATCATTaaATCATCCAGTAAACCGCC |
|  |  | CCTCGTAAATCCTCATCAaaTTAACTGGGTCAAAGGCATCATGAT |
|  |  | AAAAATCCTGTACATATTCATATCAaaATCATCCAGTAAACCGCC |
|  |  | CCTCGTAAATCCTCATCAaaTACAATTATAATGGTCGCAGCAGAA |
|  |  | TGTTGCCTTTTTATTTGTAATACAGaaATCATCCAGTAAACCGCC |
|  |  | CCTCGTAAATCCTCATCAaaAAAGTGGAGGATGATGATAAAAACA |
|  |  | TGAACATAACAGTGGATATTCCCAAaaATCATCCAGTAAACCGCC |
|  |  | CCTCGTAAATCCTCATCAaaGGGAATTTGATACACATATGAACTC |
|  |  | CTGACAATTCTTGTGCGCAAGGTTCaaATCATCCAGTAAACCGCC |
|  |  | CCTCGTAAATCCTCATCAaaACATGACTTCTCTCTATTCCTCAGA |
|  |  | ATATTCAAGTAAAGTCGACAATTTTaaATCATCCAGTAAACCGCC |
|  |  | CCTCGTAAATCCTCATCAaaTATGTTCACTTGCTAACAAATATAA |
|  |  | TTTGAAGCAGCATTTCCAGGCACTGaaATCATCCAGTAAACCGCC |
|  |  | CCTCGTAAATCCTCATCAaaTGATTGAGAAAATAAAGAAGAAGTT |
|  |  | AAAAGAAGCCCTATCAGGCAGTCAGaaATCATCCAGTAAACCGCC |
|  |  | CCTCGTAAATCCTCATCAaaAACAAACATCATTAGCAAATTCTGT |
|  |  | CAACAGAATTAACCCGATCTGGTGGaaATCATCCAGTAAACCGCC |
|  |  | CCTCGTAAATCCTCATCAaaGAGTTTGTACTGTTAATTCACAAAA |
|  |  | TAAAAACATGTAGAGTGGTTGATAAaaATCATCCAGTAAACCGCC |
|  |  | CCTCGTAAATCCTCATCAaaTATTGTGTATCAGCAGGATGCAAAA |
|  |  | GAAGATACAGGCAATGATATAGGACaaATCATCCAGTAAACCGCC |
|  |  | CCTCGTAAATCCTCATCAaaCTCCATGCAGTCTCTTGAAGATTAC |
|  |  | TAGGAAATCACAATATATAGGCAAGaaATCATCCAGTAAACCGCC |
|  |  | CCTCGTAAATCCTCATCAaaATTCATAACAGCCAGTATCCCAGCC |
|  |  | CGAAAACTCAGACGGTATAGAAGGGaaATCATCCAGTAAACCGCC |
|  |  | CCTCGTAAATCCTCATCAaaTTGGGTGCTTGGCTGTCAACACAGG |
|  |  | GAACTAGCAGATGGGGATGCGAAGAaaATCATCCAGTAAACCGCC |
|  |  | CCTCGTAAATCCTCATCAaaACCATATATAGTAGCCGTAAACAAT |
|  |  | GTTTAAAACAGTTGACACGAAAACAaaATCATCCAGTAAACCGCC |
|  |  | CCTCGTAAATCCTCATCAaaTGGGACACCAAAATTTGATTATACT |
|  |  | AAAAACAAACAGCAGCATTTTAGGAaaATCATCCAGTAAACCGCC |
|  |  | CCTCGTAAATCCTCATCAaaCACAGCTTATATTTAATGATGACCA |
|  |  | ACTCCTGCAAACTTCTAAAGGAAGAaaATCATCCAGTAAACCGCC |
|  |  | CCTCGTAAATCCTCATCAaaGCTTTGAGAGCTATTCCTGCAATTT |
|  |  | ATGCGAAATGTGTTGGCTGCCCTGAaaATCATCCAGTAAACCGCC |
|  |  | CCTCGTAAATCCTCATCAaaTGACAAAATAGAAATAAATGTTGGA |
|  |  | TGTTGTCTTCCATACAGTAGCAAAAaaATCATCCAGTAAACCGCC |
|  |  | CCTCGTAAATCCTCATCAaaTGTTGTTGTAGTAGTGGGATTTTTA |
|  |  | TTATTTCAGCATCTTCTTCTACATAaaATCATCCAGTAAACCGCC |
| L | B3_BUNVL_28_Dla50 | GTCCCTGCCTCTATATCTttCAGAATCCAAGCAGCTAGAACAGCT |
|  |  | GGACCAAGCTTATGATCAATACCTGttCCACTCAACTTTAACCCG |
|  |  | GTCCCTGCCTCTATATCTttTAACTTGTTAAACTATAACATACCC |
|  |  | AATCATCCTTGATGTTGTGCCAGGTttCCACTCAACTTTAACCCG |
|  |  | GTCCCTGCCTCTATATCTttGGAATTTCTGATGATGATAGCACAT |
|  |  | GTTGCTGGACAAGTTTGCTGATGATttCCACTCAACTTTAACCCG |
|  |  | GTCCCTGCCTCTATATCTttACTATATGAGACCAACACAAGCTGA |
|  |  | CACATAAGATTTTCATGCTAGAAGGttCCACTCAACTTTAACCCG |
|  |  | GTCCCTGCCTCTATATCTttTTAGATTAGATAAAGAGAGGTGCCA |
|  |  | AAGAATATATATCTATAAGTAAAGCttCCACTCAACTTTAACCCG |
|  |  | GTCCCTGCCTCTATATCTttAGATTATATAGCTGAGAAATTCTCT |
|  |  | TTCGCTTGCCATTTCAAGCCACGTTttCCACTCAACTTTAACCCG |
|  |  | GTCCCTGCCTCTATATCTttTCCAGCTGAGAAATATATATCTTTC |
|  |  | CATCCGCAAATGAGCAATCATCGAAttCCACTCAACTTTAACCCG |
|  |  | GTCCCTGCCTCTATATCTttTATATAAACCAAATATATCTACCAT |
|  |  | TTCCCTGGTAAAGTTAATTTAAAAGttCCACTCAACTTTAACCCG |
|  |  | GTCCCTGCCTCTATATCTttAACATCACGTAATGATTGACTTAGC |
|  |  | ACTTCAATGCAAAAGGCCTTCATGAttCCACTCAACTTTAACCCG |
|  |  | GTCCCTGCCTCTATATCTttACAATCACTTGCGAAACCGTGTAGA |
|  |  | AATCTTTGATATTGGATACATCAAGttCCACTCAACTTTAACCCG |
|  |  | GTCCCTGCCTCTATATCTttTCAACATTGCAATTATAGGGCTCTG |
|  |  | AGAAGATGAGGAAGCCAATCTTGAAttCCACTCAACTTTAACCCG |
|  |  | GTCCCTGCCTCTATATCTttTTCAGTAAAGGTGTTTGATCGTCTA |
|  |  | AAAAATTCCTAATTATAAAGACTATttCCACTCAACTTTAACCCG |
|  |  | GTCCCTGCCTCTATATCTttCTGAACACGGACGAGATGATAAGTG |
|  |  | GAGAGAATATCAAAAGAGAGATGTAttCCACTCAACTTTAACCCG |
|  |  | GTCCCTGCCTCTATATCTttAAATGGAGTGCTCAAGATGTATTTT |
|  |  | AAACTCGAGATAAATGCAGACATGTttCCACTCAACTTTAACCCG |
|  |  | GTCCCTGCCTCTATATCTttCAATTATATGCAAAAACTATTAATA |
|  |  | AAAAACACGTATTCTGTATTTTATGttCCACTCAACTTTAACCCG |
|  |  | GTCCCTGCCTCTATATCTttATTATAATTATGTCCAAATTAAAAG |
|  |  | TGATCCTTGAGATGACTAATGGTCTttCCACTCAACTTTAACCCG |
|  |  | GTCCCTGCCTCTATATCTttCTGGATGATCAAATTAATGCACCAC |
|  |  | TGGATAACATTTTTCACTTACAACAttCCACTCAACTTTAACCCG |
|  |  | GTCCCTGCCTCTATATCTttTAACGCCTCGCAAATTTACAACATT |
|  |  | CAAGTGATATGCGAAGTAGATCACTttCCACTCAACTTTAACCCG |
|  |  | GTCCCTGCCTCTATATCTttGATTACAGCAGTATATTTGATAAAT |
|  |  | ATACTGTTTTCCCATAAACCAATCAttCCACTCAACTTTAACCCG |
|  |  | GTCCCTGCCTCTATATCTttAAGAGCTACCAGAGATCATTGGAAG |
|  |  | CCTCACTTGCAGAAGCGGATATCATttCCACTCAACTTTAACCCG |
|  |  | GTCCCTGCCTCTATATCTttGCACAACATAGACAATCCTCCTAAG |
|  |  | AACAACAGCAATTAAAGAAAAGATTttCCACTCAACTTTAACCCG |
|  |  | GTCCCTGCCTCTATATCTttAGAATCAGAGAGATGACCAAACTCT |
|  |  | TTAATAGGGAATGAAATCCTAATTTttCCACTCAACTTTAACCCG |
|  |  | GTCCCTGCCTCTATATCTttTTTAGCATGTCTAGATTATTTGTTT |
|  |  | AATGTTTTTAGTTTAAACATGACAAttCCACTCAACTTTAACCCG |
|  |  | GTCCCTGCCTCTATATCTttCTCATATGTCCAAAATGATGTTCTT |
|  |  | CTGACGAAGTTGCTACTGTAAAGAAttCCACTCAACTTTAACCCG |
|  |  | GTCCCTGCCTCTATATCTttGGAGATAGAATCTATGCCATTATTT |
|  |  | TGAAGTCATGGATTTCACAATTTCTttCCACTCAACTTTAACCCG |
|  |  | GTCCCTGCCTCTATATCTttTCAAAGAAGTTTTTGATTTTTCAAA |
|  |  | AGTTAGTTTCAGCAGGGGCAGATGAttCCACTCAACTTTAACCCG |
|  |  | GTCCCTGCCTCTATATCTttTATAATAAATACAATTTGTTCTATA |
|  |  | AGGGTTCTATTCAAAGAAAAACTTAttCCACTCAACTTTAACCCG |
|  |  | GTCCCTGCCTCTATATCTttGAGCCTTGGTCAATGATGATGTCAA |
|  |  | ATAAAGTCATTGCCAGTGATAGAGCttCCACTCAACTTTAACCCG |
