# Supplementary material for: Probing orthobunyavirus reassortment using Bunyamwera and Batai viruses as models
Source: PLoS Negl Trop Dis. 2025 May 30;19(5):e0013120. doi: 10.1371/journal.pntd.0013120 (PMC12169594; doi:10.1371/journal.pntd.0013120)
Supplement: S2 Table — (DOCX) [file pntd.0013120.s002.docx]

**Supplementary Table 2: rBATV HCR probes used in the study (Figures 5, 8 and 9).**

| **Segment** | **OligoPool name** | **Sequence** |
| --- | --- | --- |
| S | B4_BATVS_8_Dla80 | CCTCAACCTACCTCCAACaaATTAACTTTAAGCGTATCTACACCA |
|  |  | AGTACTTTTGACCCAGAGGTTGCATatTCTCACCATATTCGCTTC |
|  |  | CCTCAACCTACCTCCAACaaGGGAACAGGAACAGTCCAGTTCCAG |
|  |  | GTTACTGTATTTAATACAAATTTTCatTCTCACCATATTCGCTTC |
|  |  | CCTCAACCTACCTCCAACaaGATTCCTTGCCAGGTACTTACTTGA |
|  |  | ATGGTCTTACCCTCCACAGACTCAGatTCTCACCATATTCGCTTC |
|  |  | CCTCAACCTACCTCCAACaaATTGATTATAAAATCAAAAATTGTT |
|  |  | GATTCTAAAAGTGAGTGACCCGGAAatTCTCACCATATTCGCTTC |
|  |  | CCTCAACCTACCTCCAACaaGAGGAGGTTTACCTCTCATTCTTTC |
|  |  | AAAAACGGTATAACCTGGGCAGATGatTCTCACCATATTCGCTTC |
|  |  | CCTCAACCTACCTCCAACaaAAAGTATCTGGAGAAGACCATGCGC |
|  |  | CAAGGTGCAGAGGAAAGAGATGGAGatTCTCACCATATTCGCTTC |
|  |  | CCTCAACCTACCTCCAACaaTGGACAGTCAGTAAGCTGAACGAAG |
|  |  | AGATATATGGGCTTGGAAGCATCAAatTCTCACCATATTCGCTTC |
|  |  | CCTCAACCTACCTCCAACaaTTGGGTGGAAGAAGACAAATGTGAG |
|  |  | AGTCTGCACTGACTGTAGTATCAGGatTCTCACCATATTCGCTTC |
| M | B5_BATVM_23_Dla50 | CTCACTCCCAATCTCTATaaAGCCTCTCCTGTAATAACGAGATGT |
|  |  | TGTCTTATTAATCTCTTGCTGTGTAaaCTACCCTACAAATCCAAT |
|  |  | CTCACTCCCAATCTCTATaaAATAAAGTTTTACGAAATTGGATCA |
|  |  | AAAAATGACACTGGTTTATTTGCACaaCTACCCTACAAATCCAAT |
|  |  | CTCACTCCCAATCTCTATaaAAACACCACATGTCGTGTATTAGAT |
|  |  | AAGACCTTACAATTCCATGCCTGTTaaCTACCCTACAAATCCAAT |
|  |  | CTCACTCCCAATCTCTATaaATCACCCTTTCACAAATTGTGGATC |
|  |  | AAAAATGCCCGTGTTGTGGCCTAGCaaCTACCCTACAAATCCAAT |
|  |  | CTCACTCCCAATCTCTATaaTAATTATCCAGAATCAAGAAAATAC |
|  |  | TGTTACACCAATAGAGGGGACATTAaaCTACCCTACAAATCCAAT |
|  |  | CTCACTCCCAATCTCTATaaGTGTGTCTACTGATTGCTCAAAACA |
|  |  | TGGTCCAAAATAATTATGATGATATaaCTACCCTACAAATCCAAT |
|  |  | CTCACTCCCAATCTCTATaaCTCAGGCGAAATGGAGGTTAACAGC |
|  |  | AAGTTTTTGAGCATAACAGTGGCTAaaCTACCCTACAAATCCAAT |
|  |  | CTCACTCCCAATCTCTATaaATCATTATCCCCTACTCAACTTGCT |
|  |  | AATGTCACTGCCATATGTCAATGAAaaCTACCCTACAAATCCAAT |
|  |  | CTCACTCCCAATCTCTATaaACACCAGCAAAGCTTTACAAATCAA |
|  |  | AACTCACCAAATTACAAGATTTATAaaCTACCCTACAAATCCAAT |
|  |  | CTCACTCCCAATCTCTATaaATAGGTGGAACGTAATAAAATGTGA |
|  |  | ACAAGGGAGTATGTACAATAAATGAaaCTACCCTACAAATCCAAT |
|  |  | CTCACTCCCAATCTCTATaaAGAATAGAAAGGTTCCCAATAAATC |
|  |  | GGGGAATATTGTTTATCCCACAGCTaaCTACCCTACAAATCCAAT |
|  |  | CTCACTCCCAATCTCTATaaGTTTAAAAGAGCTATTTCTGAGAAA |
|  |  | CAGTTTAGATCTAGAAAGCTTAGAAaaCTACCCTACAAATCCAAT |
|  |  | CTCACTCCCAATCTCTATaaACATATATGACACAGGTCCTACTAT |
|  |  | GTGCCATTATTAAGGCGACTTATAAaaCTACCCTACAAATCCAAT |
|  |  | CTCACTCCCAATCTCTATaaAATTGCAGTTTAAAACAGTTGATAC |
|  |  | TAGAGCCTAAAATTACAGATGAATTaaCTACCCTACAAATCCAAT |
|  |  | CTCACTCCCAATCTCTATaaTGTGGTAACATACAGAAAACCAATT |
|  |  | AATGACTTAGGATCATTCTCACAAGaaCTACCCTACAAATCCAAT |
|  |  | CTCACTCCCAATCTCTATaaTTGATTATGTGTGTCATGGTGCCTC |
|  |  | CCATCATAGGCACAGGCACTGCAAAaaCTACCCTACAAATCCAAT |
|  |  | CTCACTCCCAATCTCTATaaCAATAATAATTATGAATCATGTAAA |
|  |  | AAAAGATATAATTGTGAGGAGATGCaaCTACCCTACAAATCCAAT |
|  |  | CTCACTCCCAATCTCTATaaAGATTTGGAAATAGAAGCAAAGTGT |
|  |  | GAGGTACAAATCATTTGCGGAAAATaaCTACCCTACAAATCCAAT |
|  |  | CTCACTCCCAATCTCTATaaCTTGTACAACTTTTCACAACAGATT |
|  |  | TTGACACTGTGTGCTCAATTGAAGGaaCTACCCTACAAATCCAAT |
|  |  | CTCACTCCCAATCTCTATaaAAGAAAACTTACACAATACTGTTTA |
|  |  | GGGCCAATAGAGGAGTTCCAGATTTaaCTACCCTACAAATCCAAT |
|  |  | CTCACTCCCAATCTCTATaaTAAGCACAGGGGACCAAACATCTTT |
|  |  | CAGTTGAAAAGAATGATAAAATCGAaaCTACCCTACAAATCCAAT |
|  |  | CTCACTCCCAATCTCTATaaGCTTTCTTTGGAAGCTACTTTAGTA |
|  |  | ATCAGTGTTATTTTCGAACCAATAAaaCTACCCTACAAATCCAAT |
|  |  | CTCACTCCCAATCTCTATaaATTAAAAAGAAATGAATACCTGTAC |
|  |  | ACCTATGATGTTTAAACTTAGAGATaaCTACCCTACAAATCCAAT |
| L | B2_BATVL_36_Dla50 | CCTCGTAAATCCTCATCAaaTAGGGATTGAATATAAGAATAATGT |
|  |  | ACTTTGGTCGTGAGCTATGCACAGCaaATCATCCAGTAAACCGCC |
|  |  | CCTCGTAAATCCTCATCAaaATCTGGGATGGCGATTTCCTGATTA |
|  |  | ATCCCGAATGTGACTCCGGACAACTaaATCATCCAGTAAACCGCC |
|  |  | CCTCGTAAATCCTCATCAaaCAATTAACAATCATAGGGGAGAATT |
|  |  | ATAATACGTGCCAACCCACTGACATaaATCATCCAGTAAACCGCC |
|  |  | CCTCGTAAATCCTCATCAaaTCCTAGGGTCCATGCCTAAAAGATT |
|  |  | TATCAGATCATCCTATATATCAAGAaaATCATCCAGTAAACCGCC |
|  |  | CCTCGTAAATCCTCATCAaaGATGAGTATGAGGGTTGCCCAGGAG |
|  |  | GAATGAGATTGACAAAGGCTGGGAAaaATCATCCAGTAAACCGCC |
|  |  | CCTCGTAAATCCTCATCAaaGCAGTCAAGATTGGTAATGCTCTTG |
|  |  | AAAAAGATAGATAATAAGAGACTTGaaATCATCCAGTAAACCGCC |
|  |  | CCTCGTAAATCCTCATCAaaTAATAACAGCATTGTTTTAATGGCA |
|  |  | CATTGAAAAACCGAAGATACTGGACaaATCATCCAGTAAACCGCC |
|  |  | CCTCGTAAATCCTCATCAaaAAAAACTTATTTTTGGCAATGCATT |
|  |  | GACAGCAGAGATTCTAAAGAAAATCaaATCATCCAGTAAACCGCC |
|  |  | CCTCGTAAATCCTCATCAaaCACAGTAGTCTTCTGCATTGTATGT |
|  |  | ATCTTCAGACATAAAGACTAAAAGAaaATCATCCAGTAAACCGCC |
|  |  | CCTCGTAAATCCTCATCAaaACAATTGAGAAACATTTACTTATCT |
|  |  | TTCTGCAAATGAGCAATCTAGTAAAaaATCATCCAGTAAACCGCC |
|  |  | CCTCGTAAATCCTCATCAaaCAATCATCTTAGAAATAGAGTGGAA |
|  |  | ATCACTCATCTTAGACACATCGAGAaaATCATCCAGTAAACCGCC |
|  |  | CCTCGTAAATCCTCATCAaaTTTTCAATAAAGGACAGAAAACAGC |
|  |  | AGAATCACAAAGAGTTTACATTCACaaATCATCCAGTAAACCGCC |
|  |  | CCTCGTAAATCCTCATCAaaAAAAGAAAGCTGAAGAGGAAATAAG |
|  |  | CTGGTGATGCAAAATTGAGAATCCTaaATCATCCAGTAAACCGCC |
|  |  | CCTCGTAAATCCTCATCAaaACCAGAAAACCCCCTATAAAAATGA |
|  |  | CAGATGATCTTATTGCGAATATCTTaaATCATCCAGTAAACCGCC |
|  |  | CCTCGTAAATCCTCATCAaaCTTTAAAACTACTTGAAGGGGATTG |
|  |  | TGGTCTACAAGGACATAATTAAAGAaaATCATCCAGTAAACCGCC |
|  |  | CCTCGTAAATCCTCATCAaaGGAATTTGTTTCACTTTTTAATCTT |
|  |  | AAAAACATATGTGACACATACCTGTaaATCATCCAGTAAACCGCC |
|  |  | CCTCGTAAATCCTCATCAaaCTAGATGATCAAATAAACTCTCCTC |
|  |  | TGGATAACTTACTTGACATACAATAaaATCATCCAGTAAACCGCC |
|  |  | CCTCGTAAATCCTCATCAaaAGTTGGGTTAGAAGCAGGAAACCTT |
|  |  | TGATGCACCATTGTATTTGATAGCTaaATCATCCAGTAAACCGCC |
|  |  | CCTCGTAAATCCTCATCAaaGAGAAACGATCCAGACCCAGTTTCA |
|  |  | AAAAACTTGTCCCACTGGACAAGCAaaATCATCCAGTAAACCGCC |
|  |  | CCTCGTAAATCCTCATCAaaCACCAAGGAAGTTTACTACTGTAGG |
|  |  | GTGATATGAGAAGCAGGTCATTACTaaATCATCCAGTAAACCGCC |
|  |  | CCTCGTAAATCCTCATCAaaCTATACTATATAGGTACAATTCAAA |
|  |  | GTGAAGACAAAGAACAATTTATGCAaaATCATCCAGTAAACCGCC |
|  |  | CCTCGTAAATCCTCATCAaaTCCTGCTCAATTGTTTATAGAGCAA |
|  |  | GTTTAAAGAAAGTTTGTCCATACAGaaATCATCCAGTAAACCGCC |
|  |  | CCTCGTAAATCCTCATCAaaAACTACCAGAAATTATAGGAAGAGT |
|  |  | CTTTAGCAGAATCAGACTTGATTGCaaATCATCCAGTAAACCGCC |
|  |  | CCTCGTAAATCCTCATCAaaAGCAAATCTACTACCGATATAGCAG |
|  |  | TCCCCAGCATTAGTTCTCAAGGCATaaATCATCCAGTAAACCGCC |
|  |  | CCTCGTAAATCCTCATCAaaAAACATAGACAACCCACCAAAGCAT |
|  |  | CACAGGGATACAAGACAAAATAAATaaATCATCCAGTAAACCGCC |
|  |  | CCTCGTAAATCCTCATCAaaAACACAAAGTTAAAGTTTTCATACT |
|  |  | TCTGCTATGATTACATAAAATCAACaaATCATCCAGTAAACCGCC |
|  |  | CCTCGTAAATCCTCATCAaaCTGCACTTTGATACAAGGTAACACT |
|  |  | GATGAAATCATATACATCTGTTGATaaATCATCCAGTAAACCGCC |
|  |  | CCTCGTAAATCCTCATCAaaATATGAGACTTGACTTCTTACCTCT |
|  |  | CCCTATATAACATAATATTAGGAAGaaATCATCCAGTAAACCGCC |
|  |  | CCTCGTAAATCCTCATCAaaTGGCAAACAAATCGATCCCTGAATT |
|  |  | ACAAACGAAAGAGTTTCATGGAACAaaATCATCCAGTAAACCGCC |
|  |  | CCTCGTAAATCCTCATCAaaAATTCTAGGCATGGCCTGAAGTTTG |
|  |  | GTATTTCATGCTGGAAATAAATTACaaATCATCCAGTAAACCGCC |
|  |  | CCTCGTAAATCCTCATCAaaAGTCTCTACTATAGATCATATAATA |
|  |  | AAAGAGAGCACACATATACACTTATaaATCATCCAGTAAACCGCC |
|  |  | CCTCGTAAATCCTCATCAaaTAGTATGTCACGATTGCATGTATCA |
|  |  | TGTCTTCAGTTTAAATATGACAAATaaATCATCCAGTAAACCGCC |
|  |  | CCTCGTAAATCCTCATCAaaTGATAACATTTGTAAATCAAGTATT |
|  |  | AACCCAGGAGTTATTGTCATTAAGCaaATCATCCAGTAAACCGCC |
|  |  | CCTCGTAAATCCTCATCAaaTTTCAGAGGAAATAGAATCCATGCC |
|  |  | TCTCAGATGAAATTATGGATTTTACaaATCATCCAGTAAACCGCC |
|  |  | CCTCGTAAATCCTCATCAaaTTCAGAGAAGATGACATATAAAAAT |
|  |  | ATTTACAATAAAATACCAGAAGAAAaaATCATCCAGTAAACCGCC |
|  |  | CCTCGTAAATCCTCATCAaaAAGTAGTGAACCGTGGTCAACCATG |
|  |  | GAAGTTTATAAAATCACTACCGGAGaaATCATCCAGTAAACCGCC |
